# It’s More than Just a Game: Exploring the Benefits of Mixed Reality on Cognition in a Stroke Case Series

**DOI:** 10.3390/jcm14227998

**Published:** 2025-11-11

**Authors:** E. Eduardo Medina, Madison A. N. Webster, Justin Huber, Amanda C. Glueck

**Affiliations:** 1 College of Medicine, University of Kentucky, 740 South Limestone Street, Lexington, KY 40536, USA; eemedina@uky.edu (E.E.M.); madison.webster@uky.edu (M.A.N.W.); 2 Department of Physical Medicine and Rehabilitation, University of Kentucky, Lexington, KY 40536, USA; justin.huber@uky.edu; 3 Department of Neurology, University of Kentucky, 740 South Limestone Street, Lexington, KY 40536, USA

**Keywords:** neurorehabilitation, mixed reality, stroke recovery, cognitive rehabilitation

## Abstract

**Background**: The chronic manifestations of stroke are commonly multisystemic, affecting motor function, perception, cognition, and more. Conventional interventions have limitations when it comes to cost and their mundane nature, which are often perceived as boring. A high prevalence of risk factors has resulted in the adult population experiencing a stroke, many of whom require medical intervention, whose limitations strain both the patient and the healthcare system. Recently, extended reality (XR) has demonstrated promise as a rehabilitative aid for cognition, proprioception, and motor function following stroke without conventional therapy constraints. **Methods**: This case series explores the relationship between mixed reality (MR; one modality of XR) and cognitive performance in three post-stroke patients. Three post-stroke participants completed 12, one-hour MR training sessions over 4 weeks. Cognitive performance was assessed and changes were compared across three timepoints: baseline, immediately following the intervention, and following a 90-day washout period. **Results**: Participants demonstrated improvement in memory, executive function, and processing speed. Additionally, two out of the three participants demonstrated trends for improvement in attention and working memory. **Conclusions**: While these promising results tentatively suggest that 12 h of mixed reality training may yield cognitive improvement in post-stroke patients, a larger sample size is needed before drawing definitive conclusions.

## 1. Introduction

According to the 2021 Global Burden of Disease (GBD), stroke incidence rose by 70% while stroke deaths rose 44% between the years of 1990 and 2021, respectively [1]. Due to the high prevalence of risk factors such as high Body Mass Index (BMI), hypertension, diabetes, smoking rates, and increasing global temperatures, stroke presents as a leading medical emergency globally. Not only does this prevalence cause a strain on global healthcare systems from a quantity standpoint, but it also demonstrates a financial significance, with an estimated global cost of $890 billion U.S. dollars accounting for 0.66% of the global Gross Domestic Product (GDP) [1]. In analyzing where these costs specifically lie, the cost models within the United States and Europe can be referenced. In Europe, for example, over a single year, it is estimated that informal care attributed 1.3 billion euros (approximately $1.5 billion US dollars) to the total cost, which directly translates to the care directly provided by families and friends as opposed to paid healthcare workers. Direct healthcare costs contributed 27 billion euros (approximately $31.8 billion US dollars), while losses due to a lack of productivity contributed 12 billion euros (approximately $14.1 billion US dollars). In contrast, indirect costs amounted to a higher contribution within the United States models, amounting to $103.5 billion dollars making up 66% of total costs. Further contributions were made with productivity loss at $38.1 billion and premature death-associated costs at $30.4 billion [2]. The significant presence of informal costs can perhaps be explained by the current inefficiency of rehabilitative systems or rehabilitative practices.

Despite the fact that there is a significant financial contribution relating to stroke and stroke recovery, studies show that patients often do not receive sufficient rehabilitation therapy following a stroke, especially if they present with mild to moderate deficits [3]. Recent studies highlight access limitations as a global issue, especially when considering factors such as socioeconomic status and geographical location [4]. This could be attributed to a multitude of reasons, including the significant cost burden seen with rehabilitation therapy, considerable time investment and transportation required to transport patients to rehabilitation sessions, and the unengaging nature of current rehabilitation therapy [5]. These reasons all contribute to the overarching phenomenon where there is a lack of current therapies with an ease of access.

The effects of stroke are multisystemic, and the most often overlooked, especially when considering rehabilitation therapy, is the cognitive impact. About 60% of stroke survivors present with cognitive impairment during their first year following a stroke [2]. These impairments can vary, including, but not limited to, memory loss, attention difficulty, language deficits, and even progress to stroke associated dementia, commonly seen as “vascular dementia [6].” Additional reviews have demonstrated that these cognitive impairments can persist long term, which further emphasizes a need for appropriate cognitive rehabilitation [7].

Cognitive dysfunction is typically identified through cognitive domains such as attention, memory, language, and executive function. Cognitive therapies thus employ systems that target these cognitive domains. Current therapies are commonly accessed through computer systems, psychosocial education, pen and paper assessments, as well as behavioral therapies [8,9]. These therapies present similar limitations to the ones previously mentioned, especially when considering from an engagement standpoint and generalizability to the real world [9]. Engagement is commonly associated with compliance when it comes to rehabilitation methods. Inevitably, employing methods such as standardized computer programs and pen and paper techniques provides a relatively mundane experience. When considering the other rehabilitative obligations stroke patients commonly have, it can be easy to see how compliance would falter in the cognitive training. These methods are also not direct translations to how patients use cognitive domains in their everyday lives. They present a one-dimensional representation of a much more complex use of cognitive function in the real world. These limitations further restrict patients from receiving and completing appropriate therapeutic interventions, especially from a cognitive standpoint. The modest benefits seen with limited applicability not only explain the restriction in patients receiving therapy, but also why they do not maintain compliance with it [10].

The concept at the heart of these rehabilitation therapies is neuroplasticity. Neuroplasticity describes organizational changes in the neuronal components within the central nervous system [11]. Much of this process occurs naturally throughout someone’s lifespan, but it can also be seen through adaptive necessity, such as the tissue death seen in strokes [11]. In order for rehabilitative practices to achieve proper neuroplasticity, certain parameters must be met. For example, not only must a specific function be trained directly, but it must also be trained with sufficient repetition. This increases the likelihood of lasting neuronal changes. Additionally, important in the initiation of neuroplasticity is the intensity of the therapy, with higher intensity more often inducing neuroplastic changes. The rehabilitative practice must also have the capacity to be implemented early as an intervention; this is especially critical following stroke-related brain damage. An important feature that seems to be lacking in current therapies is the promotion of engagement and motivation in the patient with an understanding as to why the therapy is relevant and meaningful. Lastly, the therapy must be transferable across multiple domains. For example, therapy targeting working memory must also be able to improve executive function [12].

Novel therapies aimed at improving ease of access and providing engagement incentives have shown significant promise within the neurorehabilitation space. One example is the use of extended reality (XR). XR is an all-encompassing term that includes technologies that combine both the virtual and the physical world [13]. A subset of XR is virtual reality (VR), which is further subdivided into standard VR vs. immersive VR. Standard VR presents with a completely virtual platform; however, presented within a 2D space, similar to playing a standard video game [14]. Immersive virtual reality is applied using a VR headset, where patients are completely visually (and sometimes auditorily) immersed in the virtual environment with the creation of engaging and stimulating tasks [14]. VR has shown effectiveness in cognitive rehabilitation, specifically, without the limitations of conventional rehabilitation across multiple studies [15]. Additionally, studies suggest validity with improvement of cognition with the use of VR while increasing adherence and involvement [9]. There is even the possibility of improved outcomes when compared to traditional rehabilitative methods [9]. Other facets also appear to be improved with VR. For one, outside of the cognitive analysis, it has allowed for versatility in motor deficit improvement due to its ability to display varying environments. Additionally, it has allowed for the personalization of rehabilitation, again supported by its adaptability and its ability to cater to the patient’s needs [6]. While initial integration of the systems within rehabilitation spaces would present higher up-front costs, longer-term care would prove cost-effective, especially when considering home integration and reduced lengths of hospitalization [15].

A platform that can potentially provide further improvement is a similar system known as mixed reality (MR). MR functions similarly to VR, but instead of a completely virtual environment, virtual elements are superimposed over a real-world viewpoint. This potentially addresses some of the limitations of VR. What is inevitably seen with patients who have suffered a stroke is additional incapacities, such as motor deficits. Considering this, providing therapy in a completely virtual environment may present safety concerns and mobility challenges. MR has the ability to address this while also integrating real-world applications that could improve the rehabilitation process. In theory, the combination of repetitive practice, real-time feedback, and increased safety measures provides a space ripe for rehabilitative benefit [16]. Therefore, the purpose of this case series is to explore the potential cognitive benefits of MR game training for individuals who have suffered a stroke.

## 2. Materials and Methods

This was a small-scale feasibility, interventional study where participants were recruited to complete 12, 1 h MR intervention sessions across a 4-week time period to determine the effects of MR on cognitive performance following stroke.

### 2.1. Institutional Review Board and Informed Consent Statements

All experimental protocols were approved by the institutional review board at the University of Kentucky (protocol # 43449, approved 5 January 2019). All procedures were carried out following the rules of the Declaration of Helsinki of 1975 (revised 2013). Informed consent was obtained from all participants prior to enrollment, and this case series has not been previously reported in the literature.

### 2.2. Participants

A total of 3 participants (Participant A-C) were included in the case series (see Table 1 for details). Participants were considered for inclusion in this study if they had experienced a stroke no less than 4 months prior to enrolling in the study, no history of seizures or behavioral impairments that would prevent the safe and consistent participation in the study, not currently taking opioids for pain management (as this could affect balance while using the MR headset), being able to consent for participation (having a diagnosis of mild cognitive impairment or no diagnosis of cognitive impairment), and not having a change in spasticity medication within 3 months of enrolling (this was included because motor function was assessed for some participants, but will not be presented here).

Participant A was a 31-year-old right-handed, Caucasian, non-Hispanic male with residual left hemiparesis 15 years post right MCA stroke. Participant B was a 61-year-old, Caucasian, non-Hispanic female with right spastic hemiparesis 10 years after a hemorrhagic stroke of basal ganglia. Other deficits included aphasia with word-finding difficulty and foot drop. As a result of the foot drop, Participant B completed the intervention while seated in an office chair with wheels for safety. Participant C was a 50-year-old, Caucasian, non-Hispanic female 5 months post-inferior medulla ischemic stroke, causing left hemisensory deficits, reduced temperature perception, and dysphagia. Participant A had prior experience with MR, as he had participated in a small pilot trial two years prior to enrolling in this study. Participants B and C had no prior experience with MR.

### 2.3. Mixed Reality (MR) Platform

For this study, the MR gaming program was delivered via the Microsoft HoloLens 2 (1 Microsoft Way, Redmond, WA, USA) [17]. As previously detailed in Glueck and Han [18], the HoloLens is a self-contained holographic computer that superimposes high-definition, virtual holograms across the user’s visual field through a series of clear screens via a head-mounted display. This allows the user to maintain their real-world reference while interacting with three-dimensional virtual stimuli. This is a unique feature that distinguishes this technology from other extended reality headsets and potentially offers a safer and better-tolerated delivery for programs.

The program used for the intervention in this study was RoboRaid version 1 (1 Microsoft Way, Redmond, WA, USA). RoboRaid is a first-person shooter action game that was developed by Microsoft. During gameplay, the user must eliminate or “blast” virtual opponents while avoiding “enemy fire,” while the levels of play become progressively more difficult. Blasting required participants to bring their pointer finger and thumb together while targeting the opponent. For a more detailed description of the game, see Glueck and Han [18]. To successfully navigate the game and move through the various levels of play, participants needed to be able to discriminate between virtual opponents and recall the number of “strikes or hits” needed to destroy the various types of robots that they encounter. Furthermore, the participants must also move through the 3-dimensional playing field of the real-world, which makes the game unique to other extended reality platforms.

### 2.4. Outcome Assessments

Cognitive performance was determined using the NIH ToolBox, version 2 (V2). This study used a selection of the cognitive assessments for memory, attention, working memory, executive function, and processing speed (see Table 2 for a description of each assessment).

### 2.5. Procedure

After consenting, participants completed a baseline neurocognitive assessment using the tablet-based NIH Toolbox. The assessment was conducted in a quiet, well-lit room, with the participant and an experimenter seated next to each other at a table. The tablet (and iPad) was placed within a comfortable reaching distance from the participant, and the experimenter guided the participant through the five cognitive domain assessments (see Table 2). The cognitive assessment took roughly 30–40 min to complete, and after which the experimenter scheduled the intervention sessions and the immediate post-assessment with the participant.

The intervention consisted of 12 1 h intervention sessions distributed across 4 weeks, with 3 intervention sessions scheduled each week. This interval was selected as it mimics the typical outpatient rehabilitation sessions used at the experimenter’s home facility, without posing too much burden on the participant. Participants selected 3 days of the week as well as the time of the sessions between 8:00 am and 6:00 pm. Furthermore, the total duration of the intervention of 12, 1 h intervention sessions was selected based on two factors. First, in a previous study from our lab, we used a total of an 8 h intervention distributed across an average of 35 days for a sample of 14 nonclinical participants [18]. Further examination of this study’s in-game performance data (i.e., the number of games played per session, end game scores, and each session’s highest score achieved), demonstrated that participants failed to reach asymptotic performance. Author A.C.G. interpreted this data to suggest that a longer total duration for the intervention may be beneficial for future clinical studies. Second, a review of the 38 published extended reality rehabilitation literature from 2002 to 2021 revealed a wide range of total intervention durations ranging from 15 min to 40 h, with an average intervention time of 12.06 h (SD = 8.68). Based on this information, the total training duration of 12 h was selected.

Each of the 12 intervention sessions took place in a dimly lit, quiet, relatively empty room. We have found through repeated pilot testing that lower light levels help to accentuate the 3-dimensional holographic images from the MR headset and improve the game training experience for participants. Even though the HoloLens allows the participant to maintain their visual awareness of the real-world environment, we have found that participants may still bump into objects during the intervention session; therefore, we elected to utilize a relatively open area to mitigate the risk of falling. In instances where participants may have a balance impairment or lower extremity mobility impairments, we allow participants to remain seated during game training in a wheeled office chair with armrests. We have elected to employ this approach out of an abundance of caution and because unpublished pilot testing has revealed similar motor, balance, and cognitive benefits in both nonclinical and clinical participants. Prior to the start of each intervention session, participants were reminded that they could rest whenever they needed to and were provided with water during breaks.

**Figure 1:**
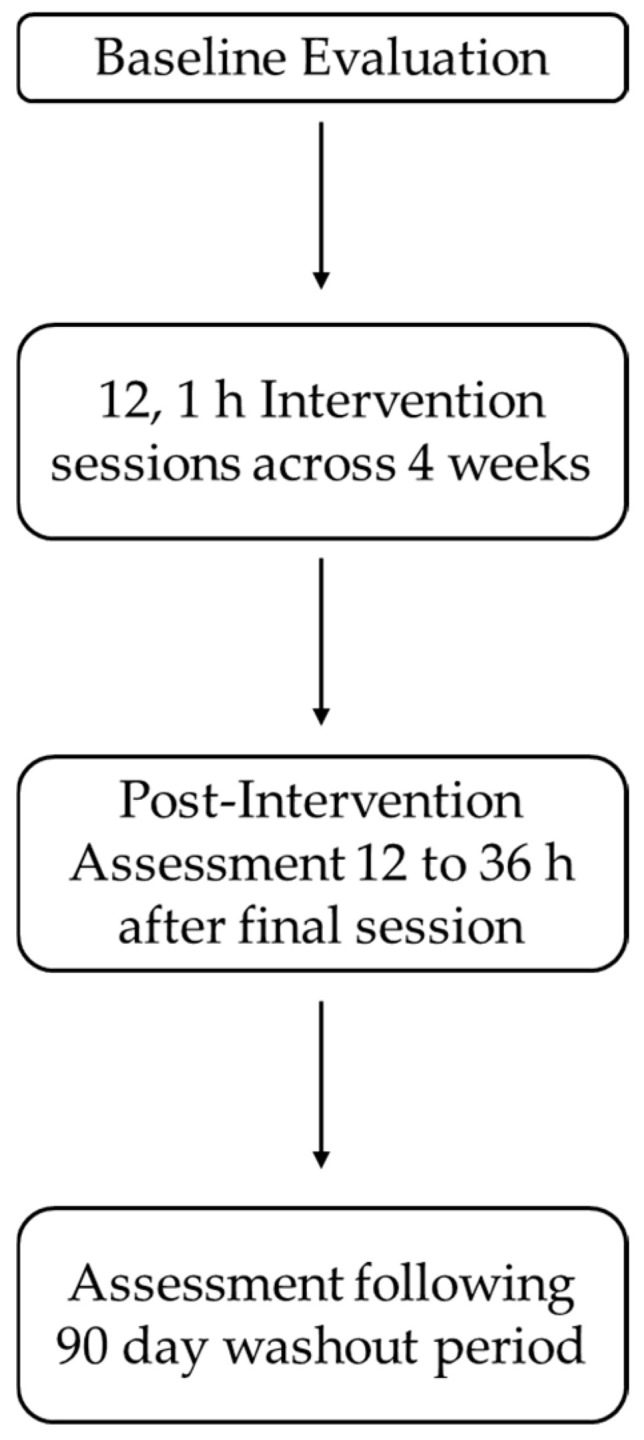
Outline of study timeline.

Upon the completion of 12 intervention sessions, participants returned for the immediate post-intervention assessment between 12 and 36 h. The immediate post-intervention involved a repeat of the baseline assessment. We employed this post-intervention assessment window to ensure any transient effects of game play were eliminated as potential confounds for the cognitive assessments rather than the effects of MR intervention itself [16,19]. Prior to leaving the immediate post-intervention assessment, participants scheduled their 90-day follow-up assessment, which was a repeat of the immediate post-intervention assessment. We employed this wash-out period because we were interested in learning whether any cognitive performance gains observed during the immediate post-intervention assessment would be stable for our participants or whether performance would revert to baseline following a 90-day washout period (Figure 1).

## 3. Results

For the presentation of the results for each participant, we elected to use the NIH ToolBox’s age-corrected standard score normalizes each participant’s raw score against a national sample of individuals of the same age band [20]. The mean for the age-corrected standard score (ss) is 100 with a standard deviation of 15. The use of these scores allows for the comparison across other studies and is frequently used in author ACG’s research as a comparison to non-clinical, healthy participants.

### 3.1. Participant A

**Figure 2:**
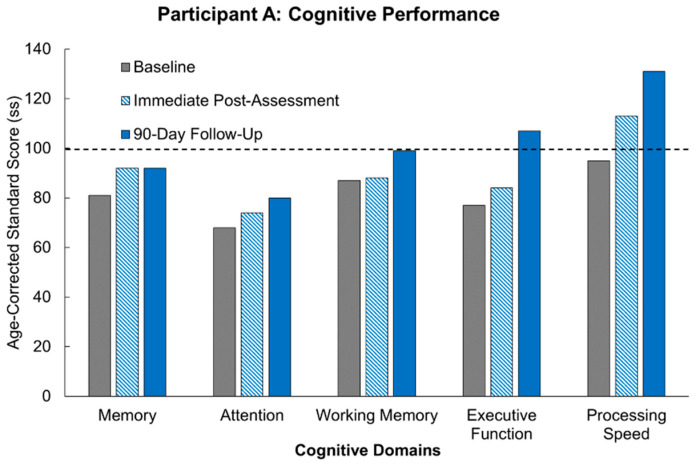
Scores for Participant A compared to age-corrected normative average in five cognitive domains and across three timepoints (baseline, immediate post-intervention assessment, and following a 90-day washout period). The dotted line represented the average age-corrected standard score for the participant’s age group.

Participant A’s scores are outlined in Table 3 and illustrated in comparison to age-matched controls in Figure 2. This participant’s baseline scores were minimally 1 standard deviation lower than age-matched controls in the domains of memory (ss = 81), attention (ss = 68), working memory (ss = 87), executive function (ss = 77), and just below average for processing speed (ss = 95). Immediately following the intervention, Participant A showed improvements in attention (ss = 92), executive function (ss = 84), processing speed (ss = 113), and memory (ss = 92). In the instance of processing speed, this improvement was over a standard deviation, and brought his scores almost a standard deviation above age-matched controls. Following the 90-day washout period, Participant A maintained improvement in memory (ss = 92) and demonstrated even further improvements in executive function (ss = 107), attention (ss = 80), processing speed (ss = 131), and working memory (ss = 99). For executive function and processing speed, Participant A was able to achieve scores higher than the age-corrected normative average, which represents improvements of 2.0 and 2.5 standard deviations (respectively) above his baseline scores.

### 3.2. Participant B

**Figure 3:**
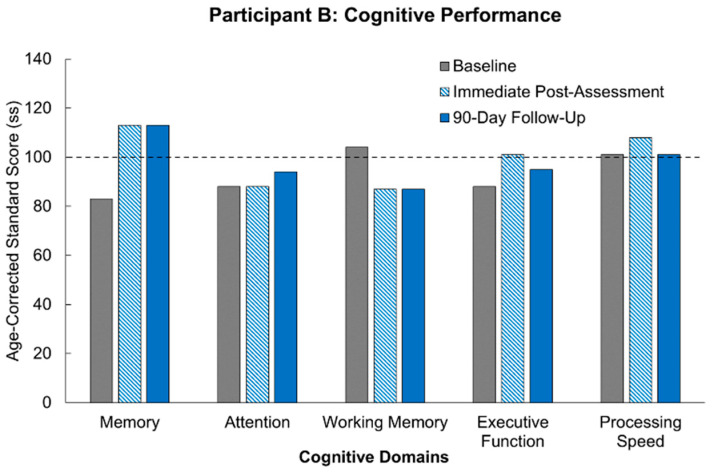
Scores for Participant B compared to age-corrected normative average in five cognitive domains and across three timepoints (baseline, immediate post-intervention assessment, and following a 90-day washout period). The dotted line represented the average age-corrected standard score for the participant’s age group.

Participant B’s scores are outlined in Table 4 and illustrated in comparison to age-matched controls in Figure 3. Participant B’s baseline scores were minimally 1 standard deviation lower than age-matched controls in the domains of memory (ss = 83), executive function (ss = 88), and attention (ss = 88). Immediately following the intervention, Participant B’s scores in processing speed (ss = 108), memory (ss = 113), and executive function (ss = 101) showed improvement. For the domain of memory, this improvement was 2 standard deviations above her baseline score, indicating a significant increase in performance. For the domain of executive function, this improvement was just below a standard deviation, and for the domain of processing speed, this change was roughly 0.5 standard deviation. Her attention score remained unchanged from her baseline performance. Following the 90-day washout period, improvements were sustained in domains of executive function (ss = 95) and memory (ss = 113), and in the case of attention (ss = 94), there was nearly a 0.5 standard deviation improvement. Her processing speed score returned to baseline (ss = 101). Her processing speed score met the age-corrected normative average, while scores in memory were able to exceed that of the age-corrected normative average. For the domain of working memory score, we noticed a notable decrease (ss = 104 to ss = 87) from baseline to post-intervention. We do not have any study-related explanation for this decrease in performance, as the intervention has previously been shown to improve cognitive performance [14]. The participant did not report any noticeable change in her working memory, but given that the decline was roughly 1.5 standard deviations, we reported it to her personal physician for monitoring.

### 3.3. Participant C

**Figure 4:**
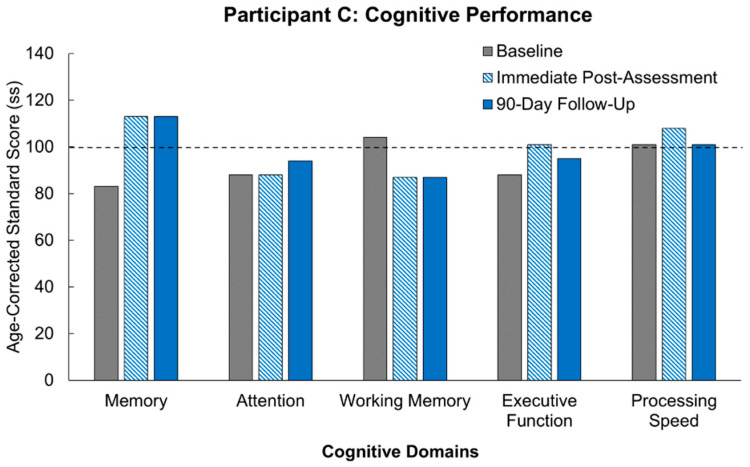
Scores for Participant C compared to age-corrected normative average in five cognitive domains and across three timepoints (baseline, immediate post-intervention assessment, and following a 90-day washout period). The dotted line represented the average age-corrected standard score for the participant’s age group.

Participant C’s scores are outlined in Table 5 and illustrated in comparison to age-matched controls in Figure 4. Participant C’s baseline scores were similar to age-matched controls (ss = 100) across all domains except attention (ss = 71), which was just under 2 standard deviations below age-matched controls. Scores immediately following the intervention, at the immediate-post assessment, demonstrated improvements across all cognitive domains tested: memory (ss = 113), attention (ss = 95), working memory (ss = 127), executive function (ss = 119), and processing speed (ss = 125). For the domain of memory, this improvement was just under 1 standard deviation. For attention and working memory, the improvements were roughly 1.5 standard deviations. The improvement in processing speed was 2 standard deviations, and in the domain of executive function, the improvement was greater than 1 standard deviation. These improvements were maintained following the 90-day washout period for attention (ss = 89), working memory (ss = 127), and executive function (ss = 113). For processing speed (ss = 138), and memory (ss = 124), Participant C demonstrated further improvements, with gains of roughly 3 and 1.5 standard deviations from baseline, respectively. Participant C’s post-intervention and follow-up scores exceeded the normative average by minimally 1 standard deviation for all domains except attention.

## 4. Discussion

As technological advances begin to integrate within rehabilitation spaces, questions arise as to their applicability towards rehabilitative efforts, such as within cognitive rehabilitation. While studies exist showing promising results using VR as a cognitive rehabilitative platform, mixed reality (MR) has yet to be fully explored within the same space. This case series was an early feasibility examination into the efficacy of using MR as a cognitive rehabilitation aid for subacute and chronic stroke patients.

Our results following MR intervention amongst our participants demonstrate the promise of the true potential of MR usage. Of significant note is the fact that all three participants demonstrated improvement across multiple domains following the MR intervention. Not only was there improvement, but for most of the cognitive domains, the participants demonstrated improvements above their own starting baselines. These results are congruent with supporting studies aligning immersive technology with subsequent enhanced neuroplastic changes [10]. At the start of the study, two out of the three participants were performing at least a standard deviation below others of their age-group who had not suffered from a stroke. What is truly remarkable for our participants is that following 12 h of our MR intervention, the participants showed cognitive recovery to the level of performance of healthy participants, and in a few domains, we actually saw the participants’ performance surpass that of healthy controls. Furthermore, these improvements were stable across 90 days of no intervention, and in some cases, the participants continued to show improvement. This sustained effect is in line with the parameters supporting neuroplasticity including task relevance, repetition, and intensity [12]. Another important aspect of our study to note is that two of our three participants were more than 10 years out from their strokes. Demonstrating continued recovery is possible for chronic patients.

What can further be observed is that the consistency of improvement across participants persists despite their varying sites of injury. In Participant B, for example, her stroke affected the basal ganglia, which is most associated with cognitive dysfunction across the three participants, yet multiple cognitive domains demonstrated improvement following intervention. In contrast, Participant C suffered injury to her brainstem following stroke, which is the location across all three participants least associated with cognitive dysfunction relatively. This explains why she tested at age-controlled baseline levels across multiple domains initially. The apparent improvement across multiple cognitive domains despite the initial baseline results further suggests efficacy when using MR gaming as an intervention.

Participant B presents notable results considering she completed the study while seated. It was mentioned prior that MR could improve upon the success of VR as a rehabilitative method as its real-world integration could present a safer modality for stroke patients who would inevitably have additional motor dysfunction. This participant experienced foot drop following her stroke and was able to complete the intervention without any complications and with demonstrated improvement across multiple domains. The success of this could be used as corroboration of the previous claim. One set of results for Participant B raised some concern. Her working memory regressed from her initial baseline at both post-intervention assessments. Up to this point it has been difficult to identify an explanation for this anomaly. However, there is no reason to assume that this result is anything more than an isolated irregularity. Despite this likelihood, there is demonstrative importance in monitoring individualized variability in response to cognitive interventions [10]. As the researchers expand the sample size, it will be a point of monitoring to see if a trend arises.

What presents as further promise amongst the results is participant scoring following the 90-day washout period. An imperative to post-stroke rehabilitation is the ability for intervention to maintain rehabilitative improvements after therapy has ceased. The characteristics driving this maintenance is highlighted by the integral parameters promoting neuroplasticity, as mentioned earlier. While not all participants maintained continued cognitive improvement following the 90-day washout period, general trends displayed maintenance of post-intervention levels. Memory displayed the most promise as it was the cognitive domain that was either improved or maintained at baseline across all participants. In the individual domains that did display decreases following washout, scores did not reach below age-corrected baseline levels, especially when considering results that helped achieve immediate post-intervention scores above standard age-corrected levels. Furthermore, as with other measures, there are minor fluctuations in performance across days within the same individual, and it is generally believed that performance fluctuations that are less than half a standard deviation are generally considered to be normal performance [21].

One benefit previously mentioned in regard to immersive technology within rehabilitation is the increased engagement factor it can provide. This factor can help maintain the parameters required for neuroplastic changes. However, it is important to note that there is inevitable variability across demographics such as age and sex, that would affect engagement levels in an MR system. For example, the elderly, who do not have advanced technological experience, would have a larger learning curve, limiting their engagement. However, author A.C.G. has conducted several extended reality studies in aging populations (with our oldest participant being 94 years of age), and the novelty of the technology has, to date, been an asset rather than a deterrent. Furthermore, in our experience, the novelty of the MR platform has been a draw to our older and younger participants alike. Specifically related to the current study, we have promising pilot feedback from participants as old as 75, to support that MR is accessible and engaging to a wide age range. Moreover, as the population continues to age, more and more individuals will have had experience with more advanced and immersive technologies. Therefore, the previously mentioned challenge of unfamiliarity will eventually become obsolete. Secondly, due to video games being more popular amongst males, there may be more initial engagement amongst males than females. This is an important consideration to have as it does present potential challenges in the full integration of an MR system. However, two factors around MR could address these challenges. For one, it has potential as a standard within the rehabilitative space. Most medical innovations begin with some unfamiliarity, especially when being first introduced to its respective sector. However, becoming standard practice inevitably creates familiarity, especially as there are proven outcomes. Secondly, MR has customizability, which would ease the transition of learning a new technology and cater to individual experience.

While the limited scope of this review cannot draw a causal relationship, it opens the door for more extensive evaluation. Thus, studies with larger samples sizes and varying periods of intervention are needed before widespread integration of MR into cognitive rehabilitation. With further experimentation, the future applicability of MR within post-stroke rehabilitation can be considered. For example, it is potential to act as an integrated telemedicine tool, considering it is a platform accessible to patients outside of a healthcare setting. Additionally, because there is versatility in the types of programs displayed by MR consoles, there is potential for rehabilitative personalization that caters to each patient’s needs.

## 5. Conclusions

Stroke is a leading cause of morbidity and mortality, leaving survivors with significant physical impairments, cognitive deficits, and financial burden. Virtual and mixed reality modalities offer opportunities for rehabilitation to be more individualized, engaging, and accessible for patients. The results of this study demonstrated that mixed reality rehabilitation can lead to cognitive improvements in post-stroke patients with different sites of primary lesion and time since stroke. The results suggest that these improvements can be maintained and, in some instances, even augmented following the completion of therapy. While promising, this study was limited by the small sample size. Future studies should aim to explore the use of mixed reality rehabilitation in a larger population.

## Figures and Tables

**Table 1:** Patient demographic, stroke site location, and co-morbidities for the three participants in the case-series. Abbreviations used in this table are as follows: years of age (yoa); Cerebrovascular accident (CVA); Middle Cerebral Artery (MCA); Arteriovenous Malformation (AVM); Deep Vein Thrombosis (DVT).

	Age	Gender/Sex	Premorbid Handedness	Time Since Stroke	Stroke Location	Co-Morbidities
**Participant A**	31 yoa	Male	Right	15 years	Hemorrhagic CVA, involving Right MCA territory	Cerebral AVM, Hypertension, intraocular hemorrhage
**Participant B**	61 yoa	Female	Right	10 years	Hemorrhagic CVA, involving Left Basal Ganglia	Hypertension, Hypothyroidism, DVT
**Participant C**	50 yoa	Female	Right	5 months	Ischemic CVA, involving Right Medulla	Carpal tunnel syndrome, Restless leg syndrome, Hypertension

**Table 2:** Description and order of NIH ToolBox assessments used for the 5 cognitive domains examined in this case series. (Adapted from https://nihtoolbox.org/domain/cognition accessed on 26 September 2025).

**NIH Toolbox Cognition Battery—Ages 18+**	**Name**	**Description of Assessment**	**Domain Examined**
	Picture sequence	Participants were asked to reproduce a sequence of pictures that is shown on the screen. The sequence was presented in the format of an oral story with accompanying images related to events in the story. Participants listened to the story and viewed the pictures before being asked to recall the sequence of the events in the story, with sequences progressively increasing in length across two trials. Form A was used for the baseline assessment, Form B was used for the immediate-post assessment, and Form C was used for the 90-day follow-up assessment.	Episodic Memory
	Flanker Inhibition and Attention	Participant focused on a centrally located stimulus while inhibiting attention to stimuli flanking it. The participant was presented with a group of arrows and must select the direction the middle arrow pointed. In some instances, all arrows point the same way, while in others, the middle arrow pointed in the opposite direction.	Executive Function and Attention
	List Sorting	Participant recalled and sequenced different presented (visually and orally) stimuli. The participant was presented with lists of objects and afterward must name the objects in order from smallest to largest based off memory. The lists progressively increase in length.	Working Memory
	Dimensional Change Card Sort	Measured cognitive flexibility and attention. Pictures were presented varying along two dimensions. One picture matched the color of the object, and one matched the shape. Participants were tasked with selecting the correct picture based on the cue word on the screen.	Executive function
	Pattern Comparison Processing Speed	Measures speed of processing. Participants discerned whether two side-by-side pictures were the same or not, with 85 s to respond to as many items as possible. Items were simple so as to purely measure processing speed.	Processing speed

**Table 3:** Age-corrected standard scores for Participant A across five cognitive domains at baseline, immediately following the intervention, and following a 90-day washout period. The change from baseline scores to the post-intervention assessments are shown in the bottom two rows.

	Memory	Attention	Working Memory	Executive Function	Processing Speed
Baseline	81	68	87	77	95
Post-Assessment	92	74	88	84	113
Follow-Up	92	80	99	107	131
Difference Baseline to Post	11 pts	6 pts	1 pts	7 pts	18 pts
Difference Baseline to Follow-Up	11 pts	12 pts	12 pts	30 pts	36 pts

**Table 4:** Age-corrected standard scores for Participant B across five cognitive domains at baseline, immediately following the intervention, and following a 90-day washout period. Change from baseline scores compared to the post-intervention assessments are shown in the bottom two rows.

	Memory	Attention	Working Memory	Executive Function	Processing Speed
Baseline	83	88	104	88	101
Post-Assessment	113	88	87	101	108
Follow-Up	113	94	87	95	101
Difference Baseline to Post	30 pts	0 pts	−17 pts	13 pts	7 pts
Difference Baseline to Follow-Up	30 pts	6 pts	−17 pts	7 pts	0 pts

**Table 5:** Age-corrected standard scores for Participant C across five cognitive domains at baseline, immediately following the intervention, and following a 90-day washout period. Change from baseline scores compared to the post-intervention assessments are shown in the bottom two rows.

	Memory	Attention	Working Memory	Executive Function	Processing Speed
Baseline	101	71	105	101	95
Post-Assessment	113	95	127	119	125
Follow-Up	124	89	127	113	138
Difference Baseline to Post	12 pts	24 pts	22 pts	18 pts	30 pts
Difference Baseline to Follow-Up	23 pts	18 pts	22 pts	12 pts	43 pts

## Data Availability

The data that supports this study is available upon request from the corresponding author [ACG].
